# Shall Medical Witnesses Receive a Proper Compensation?

**Published:** 1878-01

**Authors:** 


### Shall Medical Witnesses Receive a Proper Compensation?

Dr. Thomas J. Dills, of Fort Wayne, Ind., has had a controversy with the courts of his city, and came off second best. He was called to testify in a case of supposed rape. In regard to matters of fact he testified so far as he knew, but when asked his *opinion* in regard to certain matters, he refused to testify unless paid such equivalent for the service as he was in the habit of charging in his office. The judge sent him to jail for contempt of court. He was brought from the jail on a writ of habeas corpus, and the writ was argued before the same judge it is said, who of course sustained his former ruling, and remanded the prisoner to the sheriff. Dr. Dill, thinking it better to give his opinion than remain in jail, gave the required testimony and was released from custody. A fellow physician of Dr. Dill's was compelled to testify under similar circumstances. The Allen County Medical Society have taken steps to make this a test case before the supreme court, and have called upon other county societies for pecuniary assistance.—*The American Practitioner.*

